# Biochemical profiling of anti-HIV prodrug Elsulfavirine (Elpida^®^) and its active form VM1500A against a panel of twelve human carbonic anhydrase isoforms

**DOI:** 10.1080/14756366.2021.1927007

**Published:** 2021-05-17

**Authors:** Claudiu T. Supuran, Alessio Nocentini, Elena Yakubova, Nikolay Savchuk, Stanislav Kalinin, Mikhail Krasavin

**Affiliations:** a Neurofarba Department, Section of Pharmaceutical Sciences, University of Florence, Florence, Italy; b Viriom Inc, San Diego, CA, USA; c ChemDiv Inc, San Diego, CA, USA; d Institute of Chemistry, St. Petersburg State University, St. Petersburg, Russia

**Keywords:** Non-nucleoside reverse transcriptase inhibitor, elsulfavirine, N-acyl sulphonamide prodrug, human carbonic anhydrase, isoform selectivity, neuropathic pain

## Abstract

The non-nucleoside reverse transcriptase inhibitor VM1500A is approved for the treatment of HIV/AIDS in its *N*-acyl sulphonamide prodrug form elsulfavirine (Elpida^®^). Biochemical profiling against twelve human carbonic anhydrase (CA, EC 4.2.1.1) isoforms showed that while elsulfavirine was a weak inhibitor of all isoforms, VM1500A potently and selectively inhibited human (h) *h*CA VII isoform, a proven target for the therapy of neuropathic pain. The latter is a common neurologic complication of HIV infection and we hypothesise that by using Elpida^®^ in patients may help alleviate this debilitating symptom.

## Graphical Abstract

**Figure:**
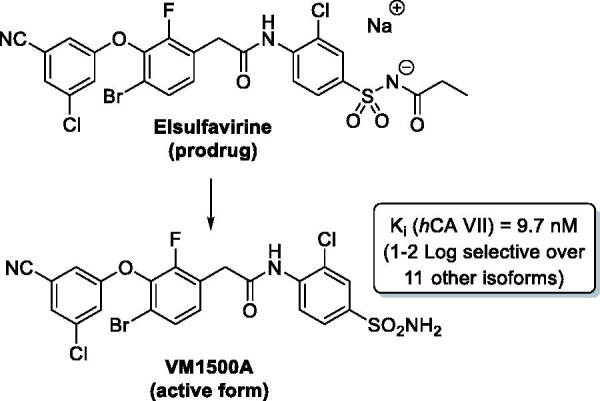

## 1. Introduction

**Figure 1.**
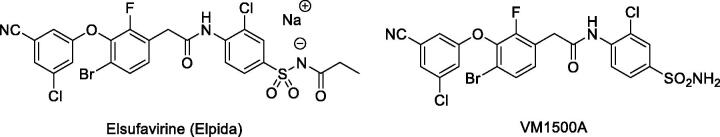
Elsufavirine (Elpida) and VM1500A.

Non-nucleoside reverse transcriptase inhibitors (NNRTIs) inhibit the human immunodeficiency virus (HIV) reverse transcriptase (RT), which is responsible for the production of double-stranded viral DNA from a single-stranded viral RNA genome^1^^,^^2^. Elsufavirine (Elpida) is a propanoyl sulphonamide prodrug form of its active form, the new-generation NNRTI, VM1500A (Figure 1).

As a drug candidate, elsufavirine was discovered by Roche. In 2009, the San Diego based Viriom biotechnology company entered into a licencing agreement with Roche for the development and commercialisation of this drug for the treatment of HIV infection^3^. Elsulfavirine demonstrated excellent antiviral efficacy in treatment-naive patients during the clinical trials. The virologic response to elsulfavirine (viral load and HIV RNA reduction) was comparable to that of the patients treated with efavirenz; however, compared to the latter, elsufavirine showed superior tolerability and fewer side effects^4^. Elsulfavirine received its first global approval on 30 June 2017, in Russia, for the treatment of HIV-1 infections in combination with other antiretroviral drugs^2^.

Elsulfavirine was shown to be rapidly converted (*t*_1/2_ ∼ 2 h) to VM1500A which is eliminated much more slowly (*t*_1/2_ > 5 days)^3^. This pharmacokinetic profile is determined by the primary sulphonamide moiety being taken up into the red blood cells (RBC). In erythrocytes, VM1500A is accumulated through reversible binding to RBC carbonic anhydrase (CA). The CA-bound drug is thereafter slowly released to plasma and ultimately reaches the target cells. This phenomenon determines the need for infrequent dosing, improves patients’ compliance and will likely improve the long-term treatment and prevention of HIV/AIDS^5^.

**Figure 2.**
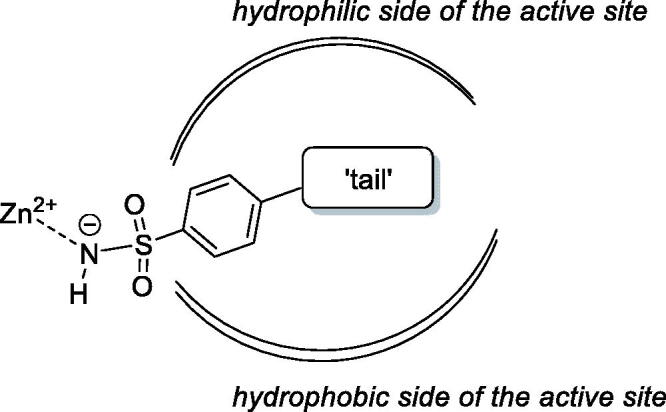
Schematic representation of a sulphonamide-based CAI bound in the active site of CA.

The primary benzene sulphonamide group ensures the affinity to various CA isoforms *via* binding, in its deprotonated form, to the zinc ion from the enzyme active site^6^. However, it is the molecular periphery (or the “tail”^7^) of the primary sulphonamide warhead that determines the differential affinity of carbonic anhydrase inhibitors (CAIs) to CA isoforms, i.e. their isoform selectivity^8^. Both polar and lipophilic periphery groups can enhance affinity to (and, hence, inhibitory potency of CAIs towards) a specific CA isoform as CAs, in general, have a very characteristic active site topology where polar and hydrophobic sides are clearly delineated (Figure 2)^9^.

The periphery of elsulfavirine and VM1500A is rather lipophilic if the tetrasubstituted diphenyl ether moiety is considered. At the same time, the benzene sulphonamide tail contains several polar moieties capable of participating in hydrogen bonding. Therefore, we were curious to establish the inhibitory profile of both agents against a comprehensive panel of recombinant CA isoforms. This, we reasoned, could help assess prospects of using elsulfavirine (VM1500A) for indications other than HIV infection/AIDS. Furthermore, considering the plethora of concomitant symptoms accompanying the advanced stages of the disease and the multifaceted therapeutic potential of isoform-selective CAIs^10^, the inhibition data obtained in multiple CA isoform inhibition assays could strengthen the position of elsulfavirine as a first-line treatment of AIDS patients. Herein, we report on the inhibitory profile of elsulfavirine and VM1500A assessed with respect to all catalytically active human (*h*CA) isoforms I, II, III, IV, VA, VB, VI, VII, IX, XII, XIII and XIV^8^.

## 2. Materials and methods

### 2.1. Compounds for biological evaluation

Active pharmaceutical ingredients elsulfavirine (Elpida^®^) and VM1500A were provided by Viriom, Inc. (San Diego, CA, USA).

### 2.2. Stopped flow CO_2_ hydrase assay

An Applied Photophysics stopped-flow instrument has been used for assaying the CA-catalysed CO_2_ hydration activity^11^. Phenol red (at a concentration of 0.2 mM) has been used as indicator, working at the absorbance maximum of 557 nm, with 20 mM Hepes (pH 7.5) as a buffer, and 20 mM Na_2_SO_4_ (for maintaining constant the ionic strength), following the initial rates of the CA-catalysed CO_2_ hydration reaction for a period of 10–100 s. The CO_2_ concentrations ranged from 1.7 to 17 mM for the determination of the kinetic parameters and inhibition constants. For each inhibitor, at least six traces of the initial 5–10% of the reaction have been used for determining the initial velocity. The uncatalysed rates were determined in the same manner and subtracted from the total observed rates. Stock solutions of inhibitor (0.1 mM) were prepared in distilled-deionised water and dilutions up to 0.01 nM were done thereafter with the assay buffer. Inhibitor and enzyme solutions were pre-incubated together for 15 min at room temperature prior to assay, in order to allow for the formation of the E-I complex. The inhibition constants were obtained by non-linear least-squares methods using PRISM 3 and the Cheng–Prusoff equation, as reported earlier^12^^,^^13^ and represent the mean from at least three different determinations. All CA isoforms were recombinant ones obtained in-house as reported earlier and their concentrations ranged between 4.1 and 9.7 nM^14–18^.

### 2.3. *In silico* studies

The crystal structure of *h*CA VII (pdb code 3ML5) was retrieved from the Protein Data Bank^19^. Сrystal structure was prepared according to the Protein Preparation module in Maestro–Schrodinger suite, assigning bond orders, adding hydrogens, deleting water molecules, and optimising H-bonding networks^20^^,^^21^. Finally, energy minimisation with a root mean square deviation (RMSD) value of 0.30 was applied using an Optimised Potentials for Liquid Simulation (OPLS-3) force field^22^. Input 3 D ligand structure was prepared by Maestro and its ionisation states were evaluated with Epik^23^. OPLS-3 force field was used for energy minimisation. Glide with default parameters was used to generate the docking grid setting the centre of the co-crystallized ligand (AAZ) as grid centre^24^. Docking was performed using the standard precision mode (SP) implemented in Glide. For each obtained docking pose, the Prime refinement was performed on all the residues located within 5 Å from the ligand^25^. Key ligand-protein interactions were analysed for the minimum-energy docking pose of VM1500A.

## 3. Results and discussion

The inhibitory profile of VM1500A, elsulfavirine and the known CA inhibitor acetazolamide (AAZ) was determined using stopped-flow CO_2_ hydrase assay as described in Section 2.2. The inhibitory data (K_i_) are summarised in Table 1.

**Table 1.** *h*CA isoform inhibitory profile of acetazolamide (AAZ), VM1500-A and elsulfavirine.

*h*CA isoform	K_i_ (nM)*		
	AAZ	VM1500A	elsulfavirine
*h*CA I	250	514	52,400
*h*CA II	12	35.5	12,500
*h*CA III	20,000	>100,000	>100,000
*h*CA IV	74	158	>100,000
*h*CA VA	63	537	4,290
*h*CA VB	54	221	14,200
*h*CA VI	11	125	5,670
*h*CA VII	2.5	9.7	2,390
*h*CA IX	25	72.1	6,650
*h*CA XII	5.7	62.3	980,1
*h*CA XIII	17	52.2	4,820
*h*CA XIV	41	15.4	1,960

*Mean from 3 different assays, by a stopped flow technique (errors were in the range of ±5–10% of the reported values).

Acetazolamide (5-acetamido-1,3,4-thiadiazole-2-sulphonamide) is known for its pan-isoform CA inhibitory profile which is considered a source of the plethora of side effects associated with this classical CA inhibitory drug^26^. Although potent and isoform-selective *N*-acyl sulphonamide CA inhibitors have been reported^27^, elsulfavirine itself demonstrated rather weak inhibition across the entire *h*CA panel, in the range of single- to double-digit micromolar concentrations with virtually no inhibition of *h*CA III and IV isoforms. To our delight, however, the primary sulphonamide VM1500A showed a much more effective inhibitory profile. The inhibition of the cytosolic *h*CA I and *h*CA II isoforms by VM1500A–abundantly present in erythrocytes and to which the drug presumably binds thus forming a slow-release depot^5^–is at the level of 513.8 and 35.5 nM, respectively. However, a notable selectivity towards cytosolic *h*CA VII isoform (generally 1–2 orders of magnitude over the majority of other *h*CA isoforms) is rather promising. *h*CA VII is a validated target for the development of inhibitors to treat neuropathic pain^28^. Considering the fact that neuropathy related to HIV is affecting as many as 50% of all individuals infected with HIV^29^–treatment of patients with elsulfavirine (Elpida^®^) may be beneficial for alleviating this most common neurologic complications of the disease.

**Figure 3.**
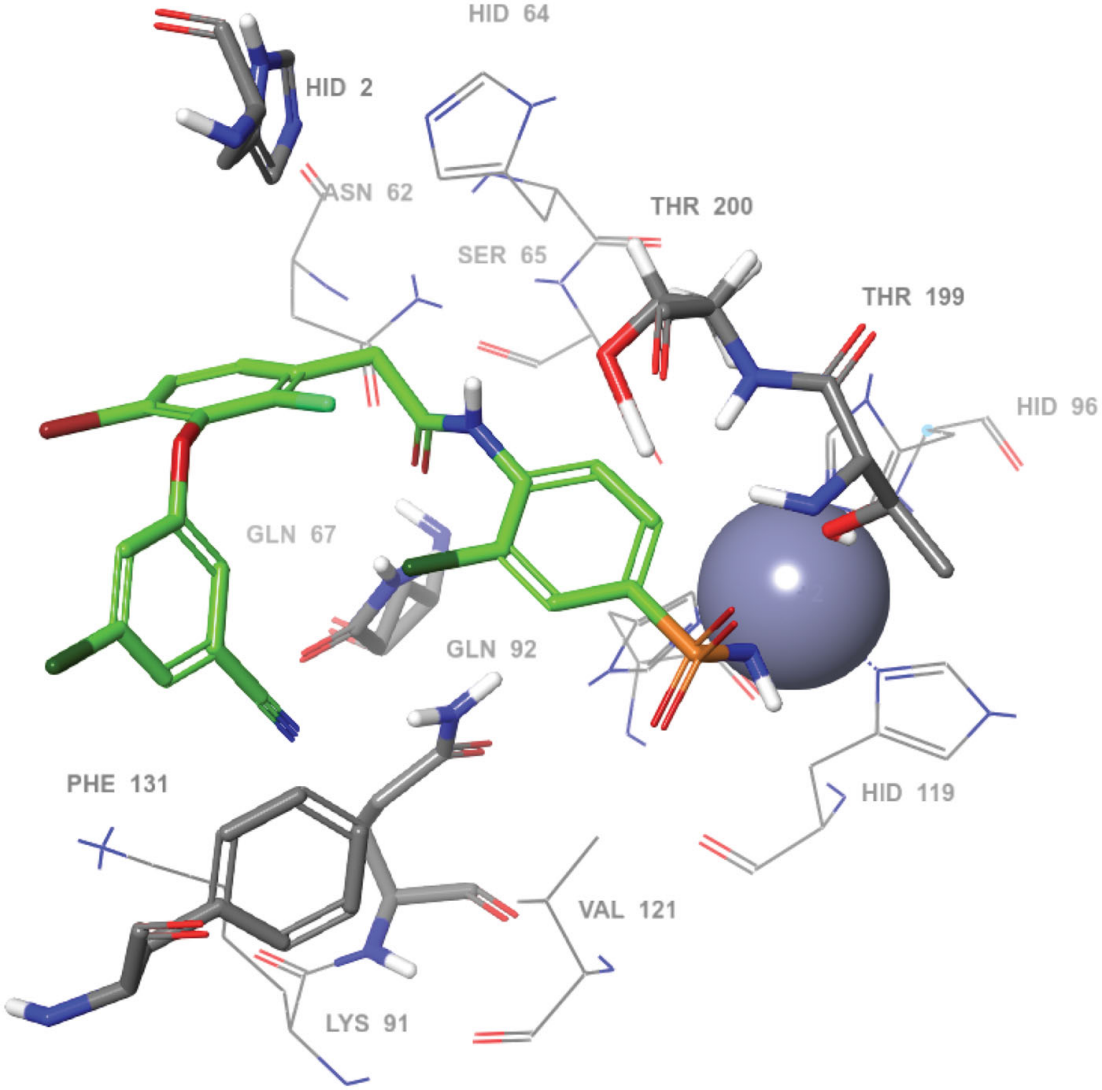
Predicted binding pose of VM1500A (green) within the active site of *h*CA VII.

Taking into account the pronounced inhibitory activity of VM1500A towards *h*CA VII isoform, we proceeded to define its likely binding mode within the active site of the protein. To this end, VM1500A was docked into the *h*CA VII crystal structure (pdb code 3ML5)^30^ and the key ligand-protein interactions were analysed for the minimum-energy docking pose shown in Figure 3. Expectedly, the orientation of the benzenesulfonamide motif was found to be the same as for the majority of such ligands in the *h*CA active site, providing anchoring to the prosthetic zinc metal ion and hydrogen bonding to the Thr199 residue at the bottom of the catalytic cavity^31^. In the meantime, the amide moiety attached to the benzenesulfonamide moiety forms contacts with amino acids Thr200, Gln67 and Gln92. Particularly, a hydrogen bond was observed between the NH group of the amide function and oxygen atom of the Thr200 side chain whereas the carbonyl group formed hydrogen bonds with NH moieties of the Gln67 and Gln92 residues. On the other hand, the ligand-protein contacts at the outer rim of the active side were mostly characterised by π–π stacking of the benzene rings with His2 and Phe131 sidechains and the bromine and chlorine substituents of these aromatic moieties were partially exposed to the bulk solvent.

## 4. Conclusion

Biochemical profiling of the new anti-HIV prodrug elsulfavirine (Elpida®) and its active form primary sulphonamide VM1500A against a panel of twelve human carbonic anhydrase isoforms revealed that while elsulfavirine had a weak inhibitory profile across the panel of *h*CAs, VM1500A showed a potent (*K_i_* = 9.6 nM) and selective inhibition of *h*CA VII, a neuropathic pain target. As neuropathic pain is common among HIV-infected individuals, concomitant inhibition of *h*CA VII may help alleviate this debilitating neurologic complication.
